# Chemical Vapor Deposition of Vertically Aligned Carbon Nanotube Arrays: Critical Effects of Oxide Buffer Layers

**DOI:** 10.1186/s11671-019-2938-6

**Published:** 2019-03-21

**Authors:** Haohao Li, Guangjie Yuan, Bo Shan, Xiaoxin Zhang, Hongping Ma, Yingzhong Tian, Hongliang Lu, Johan Liu

**Affiliations:** 1 SMIT Center, School of Automation and Mechanical Engineering, Shanghai University, Shanghai, 201800 People’s Republic of China; 2 State Key Laboratory of ASIC and System, School of Microelectronics, Fudan University, Shanghai, 200433 People’s Republic of China; 3 Shanghai Key Laboratory of Intelligent Manufacturing and Robotics, School of Automation and Mechanical Engineering, Shanghai University, Shanghai, 200072 People’s Republic of China; 4 Electronics Materials and Systems Laboratory, Department of Microtechnology and Nanoscience, Chalmers University of Technology, SE-412 96 Goteborg, Sweden

**Keywords:** Atomic layer deposition, Chemical vapor deposition, Vertically aligned carbon nanotubes, Oxide buffer layers

## Abstract

Vertically aligned carbon nanotubes (VACNTs) were synthesized on different oxide buffer layers using chemical vapor deposition (CVD). The growth of the VACNTs was mainly determined by three factors: the Ostwald ripening of catalyst nanoparticles, subsurface diffusion of Fe, and their activation energy for nucleation and initial growth. The surface roughness of buffer layers largely influenced the diameter and density of catalyst nanoparticles after annealing, which apparently affected the lifetime of the nanoparticles and the thickness of the prepared VACNTs. In addition, the growth of the VACNTs was also affected by the deposition temperature, and the lifetime of the catalyst nanoparticles apparently decreased when the deposition temperature was greater than 600 °C due to their serious Ostwald ripening. Furthermore, in addition to the number of catalyst nanoparticles, the density of the VACNTs was also largely dependent on their activation energy for nucleation and initial growth.

## Background

Vertically aligned carbon nanotubes (VACNTs) exhibit many excellent properties, including extraordinary mechanical properties, attractive electrical characteristics, and high thermal conductivity [1–3]. Therefore, VACNTs show great potential for use in a wide variety of applications, including field emitters of display, biological sensors, microelectronic devices, and hydrogen storage and thermal interface materials [4–11]. Among the existing methods, chemical vapor deposition (CVD) appears to be the most suitable for the growth of VACNTs; it offers better control of the growth parameters and the growth on predefined sites of a patterned substrate [12–17]. To achieve high-quality VACNTs by CVD, catalyst nanoparticles should be formed on and prevented from reacting with the underlying substrate [18]. Generally, to avoid undesired metal silicide formation at high process temperatures, a buffer layer is usually deposited onto the substrate prior to deposition of the catalyst [19, 20].

Many researchers have found that the buffer layer is critical to the growth of VACNTs, and different buffer layers show various effects [21]. The effective growth of VACNTs is largely dependent on the type, quality in terms of porosity, and stoichiometry of the buffer layer [22–25]. Lee et al. reported that metallic buffer layers were ineffective for the growth of VACNTs because they could not prevent diffusion of the catalyst into the substrate, resulting in the formation of carbide or silicide phases [26]. Compared with metallic films, nonmetallic films such as oxide films have been found to be more beneficial for the synthesis of VACNTs. de los Arcos et al. claimed that, compared with Al, Al_2_O_3_ resulted in more efficient growth of VACNTs when used as the buffer layer [27, 28]. In addition, compared with SiO_2_, TiO_2_, and ZrO_2_, Al_2_O_3_ was found to be a better buffer-layer material for the growth of VACNTs when Fe was used as the catalyst [29]. Although various oxide buffer layers have been introduced to increase the growth efficiency of VACNTs, their detailed role is unclear.

In this paper, we used CVD to synthesize VACNTs with different oxide films as the buffer layers. The activity and lifetime of catalyst nanoparticles were analyzed on different oxide buffer layers to achieve high-quality VACNTs. The possible growth mechanism of VACNTs is also discussed.

## Methods

Thermally oxidized SiO_2_ and three types of Al_2_O_3_ thin films were used as the oxide buffer layers. The Al_2_O_3_ films were deposited onto Si substrates by atomic layer deposition (ALD), electron-beam (EB) evaporation, and sputtering. For ALD Al_2_O_3_ films, trimethylaluminum (TMA) and H_2_O were used as the precursor and oxygen source, respectively. The deposition temperature was set at 200 °C. The thickness of the Al_2_O_3_ and SiO_2_ films used as the buffer layers was 20 nm. A 1-nm-thick Fe film was deposited onto all of them by EB evaporation; it was used as the catalyst. Afterwards, the VACNTs were synthesized by CVD (AIXTRON Black Magic II). First, hydrogen was introduced into the reaction chamber, and the pressure was set at 0.2 mbar. Before the growth of VACNTs, the catalyst was annealed at 550 °C under the hydrogen. The flow rate of hydrogen was set at 700 sccm, and the period was 3 min. Second, acetylene and hydrogen were introduced into the chamber simultaneously, and VACNTs were prepared on catalyst nanoparticles. The flow rates of acetylene and hydrogen were 100 and 700 sccm, respectively. The growth temperature was increased from 500 to 650 °C, and the growth period was fixed at 30 min.

Epoxy resin (412813) was purchased from Sigma-Aldrich Co., Ltd. The curing agent (C1486) and diluent (E0342) were purchased from TCI Chemical Industrial Development Co., Ltd. After the growth of VACNTs, VACNT/epoxy composite films were also prepared. First, epoxy resin, curing agent, and diluents were mixed as the matrix using a high-speed dispersion mixing machine (MIX500D). Second, the VACNTs were immersed into the matrix, which was subsequently cured in a vacuum oven at 120 °C for 1 h and then at 150 °C for 1 h. The obtained composite films were peeled from the Si substrate and polished to a thickness of approximately 300 μm. The tips of VACNTs protruded from both surfaces of the composite film.

Field-emission scanning electron microscopy (FESEM; Merlin Compact) was used to characterize the diameter and distribution of the catalyst nanoparticles as well as the cross section of the VACNTs and composite films. Raman spectra of the VACNTs were recorded with an inVia Reflex spectrometer, and transmission electron microscopy (TEM; Tecnai G2 F20 S-TWIN) was used to characterize the morphology of the carbon nanotubes. The chemical composition and density of different buffer layers were characterized by X-ray photoelectron spectroscopy (XPS; ESCALAB 250Xi) and X-ray reflectivity (XRR; Bruker D8 Discover), respectively. The surface roughness of different buffer layers was analyzed by atomic force microscopy (AFM; SPM9700). Laser flash thermal analysis (Netzsch LFA 447) and differential scanning calorimetry (DSC; Mettler Toledo DSC1) were used to measure the thermal diffusivity and specific heat capacity of the composite films, respectively. The thermal conductivity was subsequently calculated using Eq. 1:

1 $$ \lambda =\alpha \times \mathrm{Cp}\times \rho, $$

where *λ*, *α*, Cp, and *ρ* are the thermal conductivity (W m^−1^ K^−1^), thermal diffusivity (mm^2^ s^−1^), specific heat capacity (J kg^−1^ K^−1^), and density (kg m^−3^) of composite films, respectively.

## Results and Discussion

**Fig. 1:**
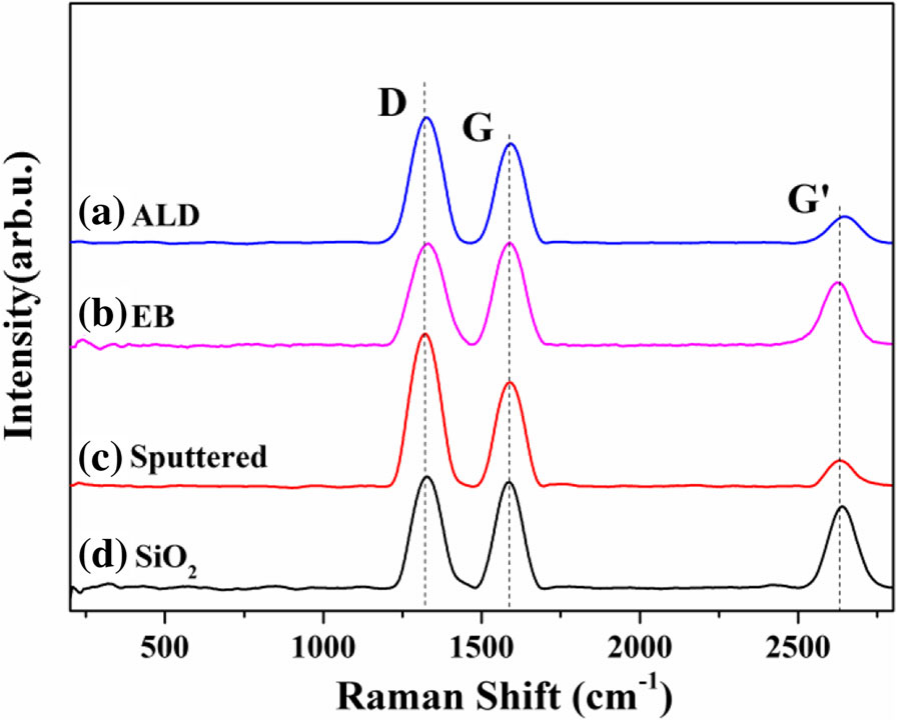
Raman spectra of VACNTs grown on different buffer layers: **a** ALD Al_2_O_3_, **b** EB Al_2_O_3_, **c** sputtered Al_2_O_3_, and **d** SiO_2_. The spectra have been normalized to the intensity of the G band to facilitate comparison

**Fig. 2:**
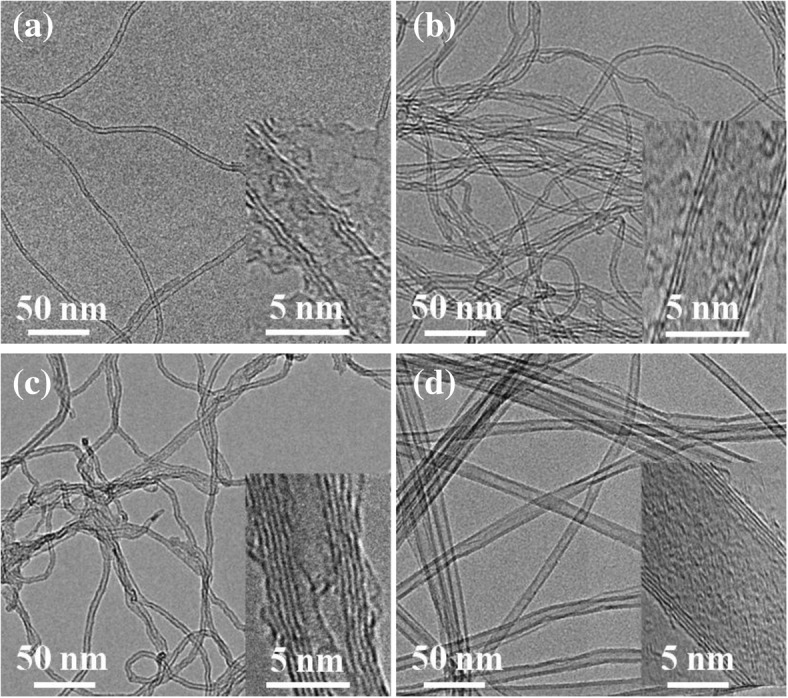
TEM images of VACNTs grown on different buffer layers: **a** ALD Al_2_O_3_, **b** EB Al_2_O_3_, **c** sputtered Al_2_O_3_, and **d** SiO_2_

Figure 1a–d shows Raman spectra of VACNTs grown on different oxide buffer layers. Generally, the G peak, which is the symmetrical vibration of the optical mode and six-ring plane expansion, was located at approximately 1580 cm^−1^ [30]. The D peak, which is a vibration mode caused by the edge or defect of the microcrystal plane, was located at approximately 1360 cm^−1^ [30]. In addition, the G′ peak was typically located at ~ 2700 cm^−1^ [31]. For different oxide buffer layers, the ratio of *I*_D_ and *I*_G_ was calculated to be approximately equal to or greater than 1, and no radial breathing modes (RBMs) were observed at ~ 200 cm^−1^. These results indicate that all of the prepared VACNTs on different buffer layers were multiwalled. Figure 2a–d shows the morphology of VACNTs on different buffer layers, which were analyzed by TEM. The VACNTs were multiwalled on all of them, consistent with the Raman analysis results. The carbon nanotubes were triple-walled on ALD and EB Al_2_O_3_ but quadruple- or quintuple-walled on sputtered Al_2_O_3_ and SiO_2_.

**Fig. 3:**
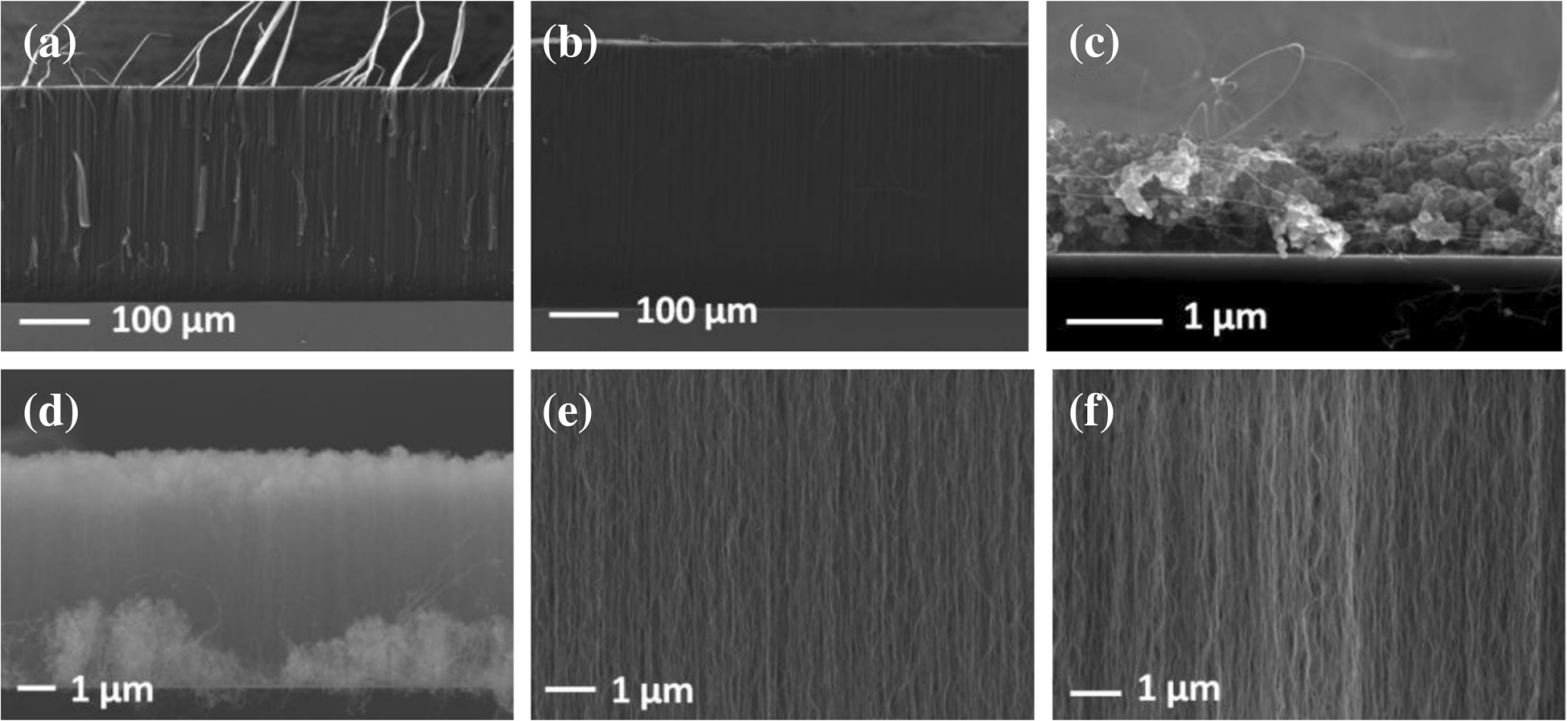
Cross-sectional SEM images of VACNTs grown on different buffer layers at 600 °C: **a** ALD Al_2_O_3_, **b** EB Al_2_O_3_, **c** sputtered Al_2_O_3_, and **d** SiO_2_. Images **e** and **f** show the internal structure of **a** and **b** at high magnification

Figure 3a–f shows the cross-sectional SEM images of VACNTs grown on different oxide buffer layers at 600 °C. The VACNTs were successfully synthesized on ALD and EB Al_2_O_3_, as shown in Fig. 3a, b, e, and f. The thickness of VACNTs on ALD Al_2_O_3_ was smaller than that on EB Al_2_O_3_, which can be explained by different lifetimes of catalyst nanoparticles on them during the growth period. The lifetime of catalyst nanoparticles, which represents the time after which the catalyst nanoparticle has basically lost its catalytic function to grow carbon nanotubes, was deduced from the thickness of VACNTs [24]. The results show that the lifetime of catalyst nanoparticles on EB Al_2_O_3_ was longer than that on ALD Al_2_O_3_, which was largely related to Ostwald ripening of catalyst nanoparticles on the substrates. Ostwald ripening is a phenomenon whereby larger nanoparticles increase in size while smaller nanoparticles, which have greater strain energy, shrink in size, and eventually disappear via atomic interdiffusion [32]. When a catalyst nanoparticle disappeared, or when too much catalyst was lost, the carbon nanotubes growing from it stopped [32]. When enough carbon nanotubes stopped growing, the growth of VACNTs collectively terminated because each terminated carbon nanotube imparted a mechanical drag force on adjacent growing nanotubes because of van der Waals forces and interlocking [32]. Therefore, the lifetime of catalyst nanoparticles was mostly dependent on their rate of Ostwald ripening. Figure 3c shows that almost no VACNTs were present on sputtered Al_2_O_3_. As shown in Table 1, the density and chemical composition of sputtered Al_2_O_3_ was almost similar to ALD and EB Al_2_O_3_, which indicated that the various Al_2_O_3_ might have a similar barrier property against Fe. Therefore, the main reason for the unsuccessful growth of VACNTs might not be the subsurface diffusion of Fe, but the serious Ostwald ripening of catalyst nanoparticles on it [33]. As Ostwald ripening proceeds, the number of nanoparticles decreases while the average catalyst diameter increases and the nanoparticle size distribution broadens [32]. Therefore, serious Ostwald ripening of catalyst nanoparticles would directly result in a low density of carbon nanotubes. Generally, any marginal alignment observed in CVD samples was due to a crowding effect, and carbon nanotubes support each other by van der Waals attraction [34]. As a result, VACNTs could not be achieved on sputtered Al_2_O_3_. Compared with VACNTs on ALD and EB Al_2_O_3_, those on SiO_2_ were very thin, which might be caused by the subsurface diffusion of Fe, as shown in Fig. 3d [33].

**Table 1:** Summary of the properties of Al_2_O_3_ films deposited by different deposition methods

	ALD Al_2_O_3_	EB Al_2_O_3_	Sputtering Al_2_O_3_
Thickness (nm)	20.00	20.00	20.00
Surface roughness (nm)	0.83	2.53	0.68
Density	2.69	2.59	2.56
Composition (%)			
Al	34.36	32.74	29.84
O	65.27	67.24	70.02
C	0.37	0.02	0.14

**Fig. 4:**
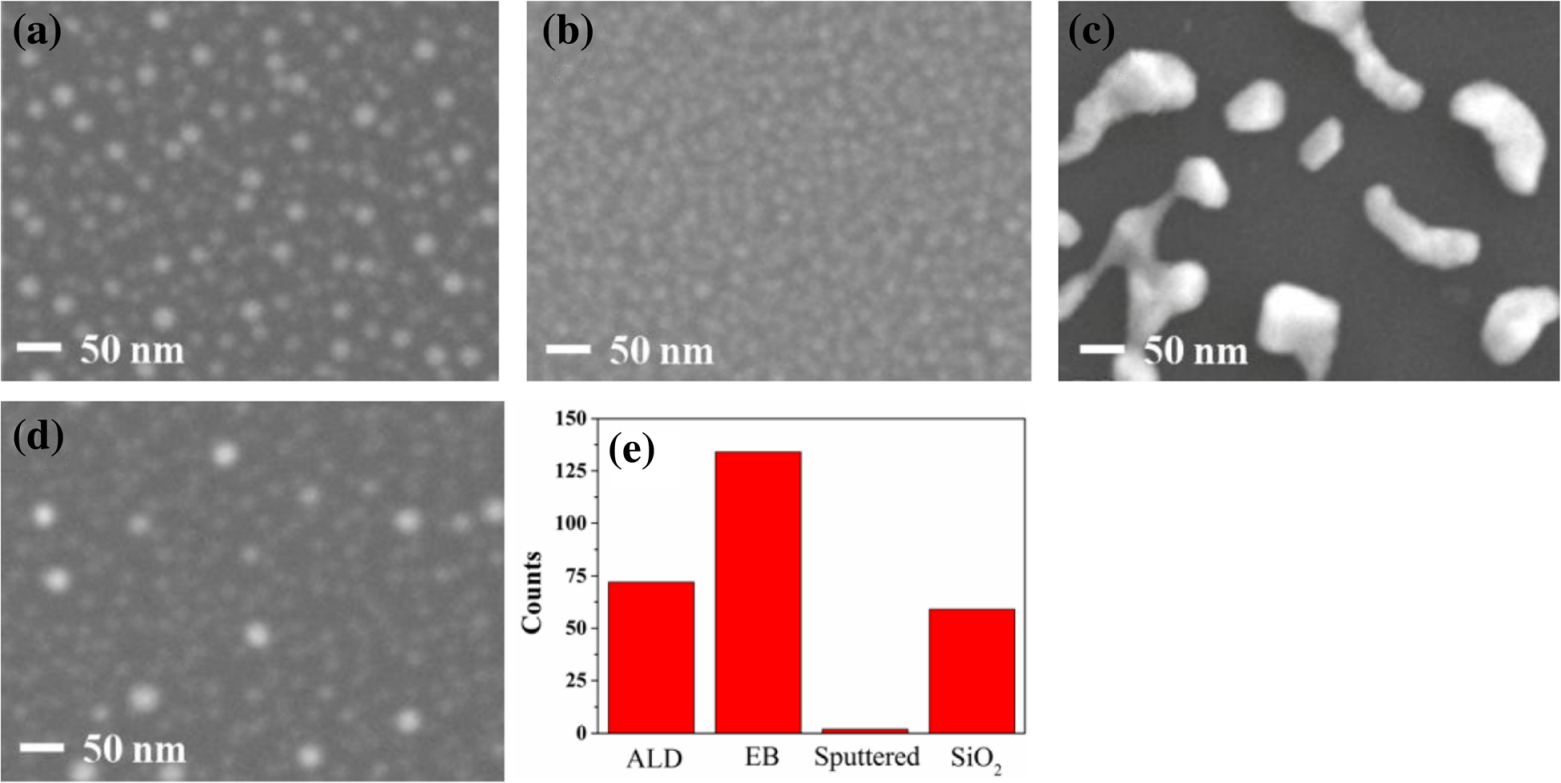
Plan-view SEM images of catalyst nanoparticles formed on different buffer layers after annealing at 550 °C in the absence of C_2_H_2_: **a** ALD Al_2_O_3_, **b** EB Al_2_O_3_, **c** sputtered Al_2_O_3_, and **d** SiO_2._ The image in **e** shows the amount of catalyst nanoparticles on a different buffer layer with a 200 × 200 nm^2^ area

**Fig. 5:**
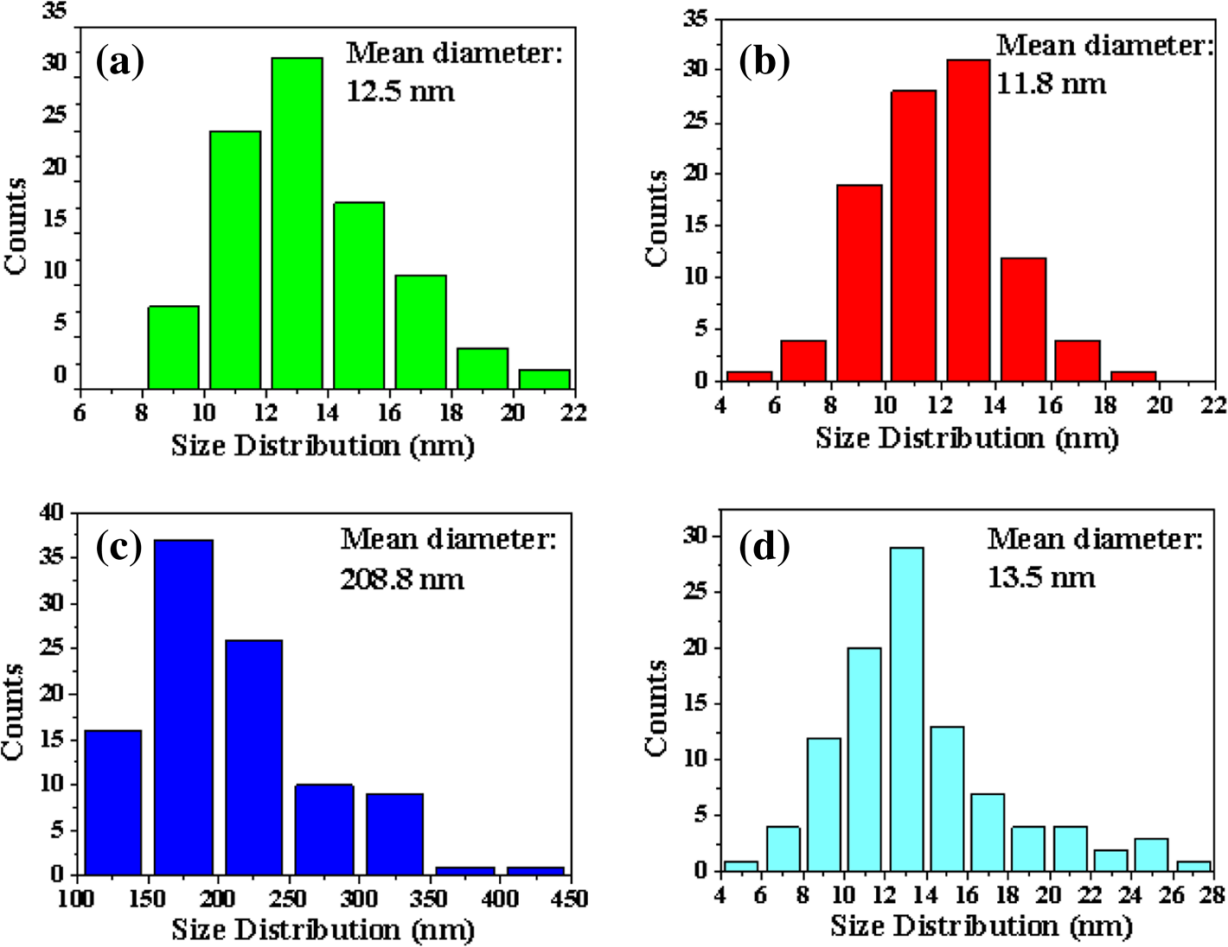
Size distribution of catalyst nanoparticles measured from the FESEM data by manual analysis of 100 particles on different buffer layers: **a** ALD Al_2_O_3_, **b** EB Al_2_O_3_, **c** sputtered Al_2_O_3_, and **d** SiO_2_

Figure 4a–d shows SEM images of catalyst nanoparticles on different oxide buffer layers after annealing at 550 °C for 3 min in the absence of C_2_H_2_. Compared with others, the nanoparticles had a much larger diameter on sputtered Al_2_O_3_ before the growth of VACNTs. Figure 4e shows the number of catalyst nanoparticles on a 200 × 200 nm^2^ area of different buffer layers. The number of nanoparticles was the most on EB Al_2_O_3_, and the least on sputtered Al_2_O_3_. The largest diameter and least number of nanoparticles might result in their shortest lifetime on sputtered Al_2_O_3_ due to the effect of Ostwald ripening. It also explains why almost no VACNTs grew on sputtered Al_2_O_3_ (Fig. 3c). In addition, the mean diameter and size distribution of catalyst nanoparticles were also analyzed, as shown in Fig. 5a–d. Figure 5b shows that the mean diameter of nanoparticles was the smallest on EB Al_2_O_3_, which led to the Fe catalyst showing the longest lifetime [35]. The result in Fig. 3b confirms that the thickest VACNTs were grown on EB Al_2_O_3_. Figure 5c shows that the mean diameter of nanoparticles was the largest on sputtered Al_2_O_3_, which was confirmed by the result in Fig. 4c. Figure 5a, d shows that the mean diameter of nanoparticles on ALD Al_2_O_3_ and SiO_2_ was similar, whereas Fig. 3a, d shows that their thickness was quite different. Fe atoms might more easily diffuse through SiO_2_ and into the Si substrate than through ALD Al_2_O_3_ [33]. The subsurface diffusion of Fe would result in few catalyst nanoparticles existing on the surface of SiO_2_ during the growth period, which led to the thin VACNTs.

**Fig. 6:**
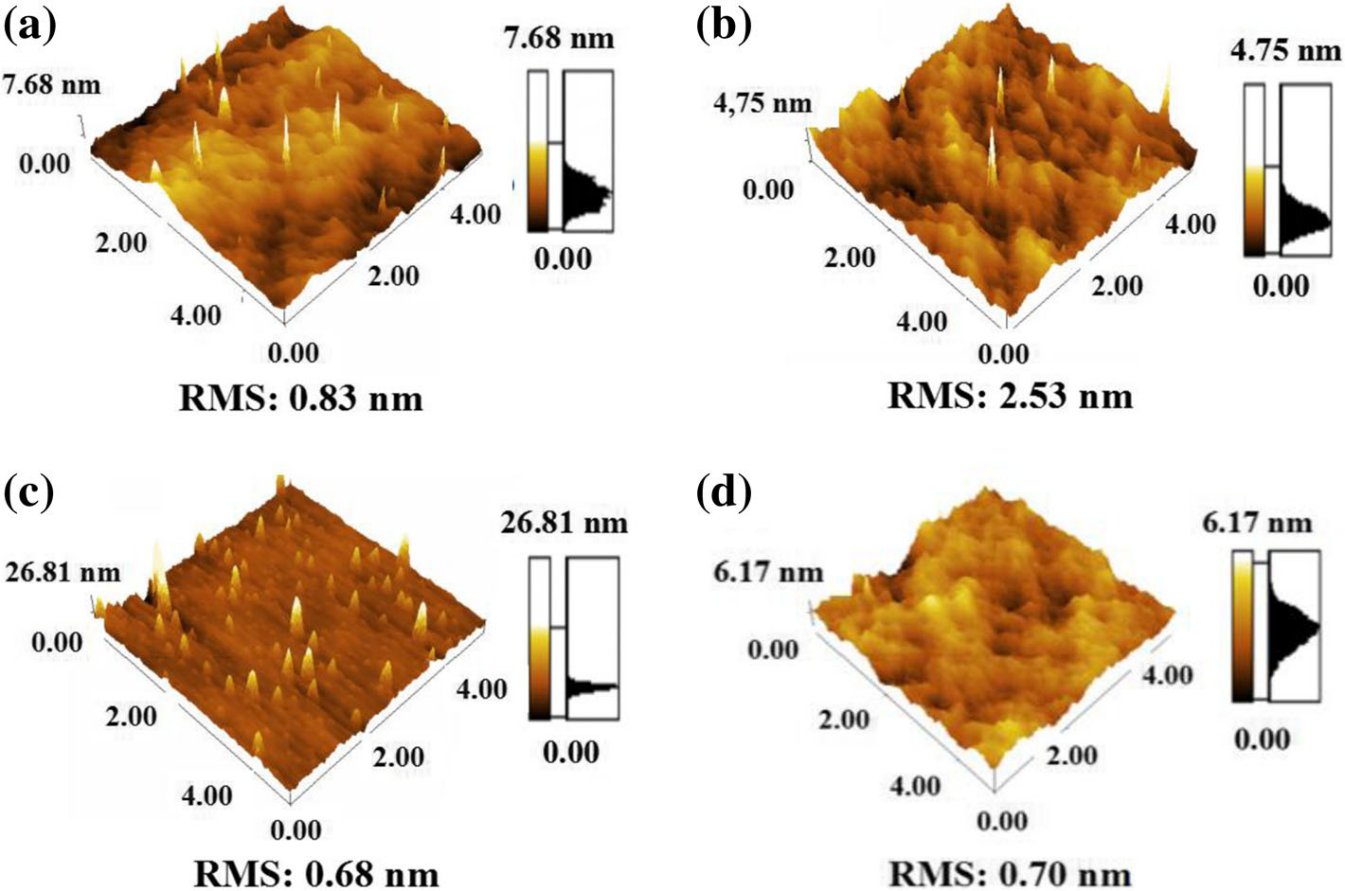
AFM topography images of the exposed buffer layers: **a** ALD Al_2_O_3_, **b** EB Al_2_O_3_, **c** sputtered Al_2_O_3_, and **d** SiO_2_

Figure 6a–d shows the surface roughness of different buffer layers before deposition of the catalyst. The surface roughness of EB Al_2_O_3_ was the largest; its root-mean-square (RMS) roughness value was 2.53 nm, as shown in Fig. 6b and Table 1. As previously mentioned, the smallest diameter and greatest number of catalyst nanoparticles were achieved on EB Al_2_O_3_. The rough surface would result in a small diameter and high density of catalyst nanoparticles after annealing. Figure 6c shows that the surface of sputtered Al_2_O_3_, whose RMS value was 0.68 nm, was the smoothest. This result indicates that the largest diameter and lowest density of nanoparticles might also be related to the smooth surface of sputtered Al_2_O_3_. From Fig. 6a, d, the RMS value of ALD Al_2_O_3_ was larger than that of SiO_2_. Compared with the nanoparticles on SiO_2_, those on ALD Al_2_O_3_ exhibited a greater density and smaller diameter, as confirmed by the results in Figs. 4e and 5a, d. Therefore, the surface roughness of buffer layers was critical and strongly influenced the growth of VACNTs in the CVD process.

**Fig. 7:**
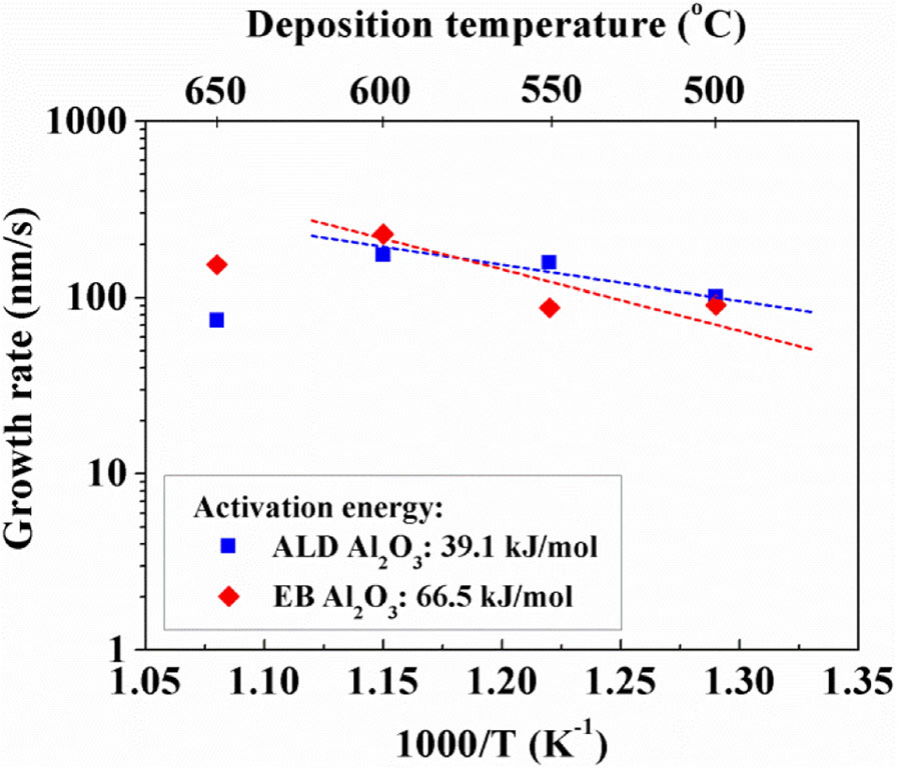
Variation of the growth rate on ALD and EB Al_2_O_3_ buffer layers as a function of the deposition temperature. The activation energies were calculated from a linear interpolation of the slopes

Figure 7 shows the effect of deposition temperature on the growth rate of VACNTs on EB and ALD Al_2_O_3_. At temperatures below 600 °C, the growth rate increased with increasing temperature. However, when the temperature was greater than 600 °C, the growth rate apparently decreased. This behavior might be related to serious Ostwald ripening of catalyst nanoparticles, which largely reduced the lifetime of nanoparticles and the growth rate [32]. In addition, Fig. 7 also shows the dependence of the growth rate on 1/T; the activation energy was directly calculated from the slope of the linear fit to the data [36]. The activation energies for the nucleation and initial growth of VACNTs on ALD and EB Al_2_O_3_ were 39.1 and 66.5 kJ mol^−1^, respectively. This result indicates that activation energy for nucleation and initial growth using ALD Al_2_O_3_ is much lower than that using EB Al_2_O_3_. Therefore, we could conclude that the nucleation and initial growth of VACNTs were more easily achieved on ALD Al_2_O_3_, compared with EB Al_2_O_3_. From Table 1, we could know that there were some impurities in ALD Al_2_O_3_, such as carbon, which might offer the extra sites for the nucleation of VACNTs and then reduce its activation energy.

**Fig. 8:**
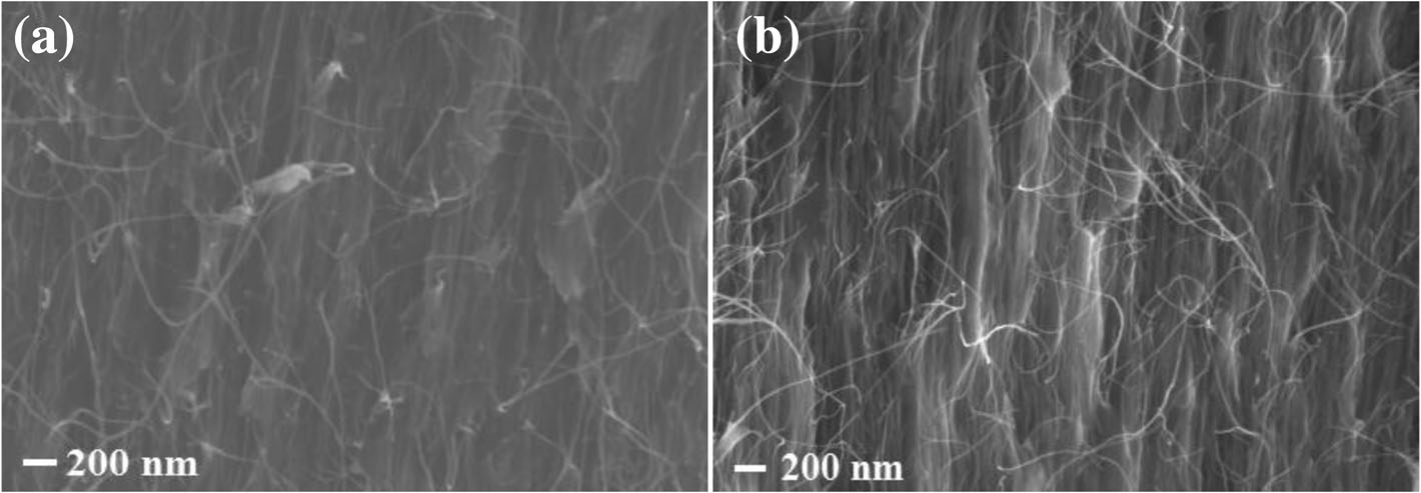
Cross-sectional SEM images of composite films with VACNTs grown on different buffer layers: **a** ALD Al_2_O_3_ and (**b**) EB Al_2_O_3_

**Fig. 9:**
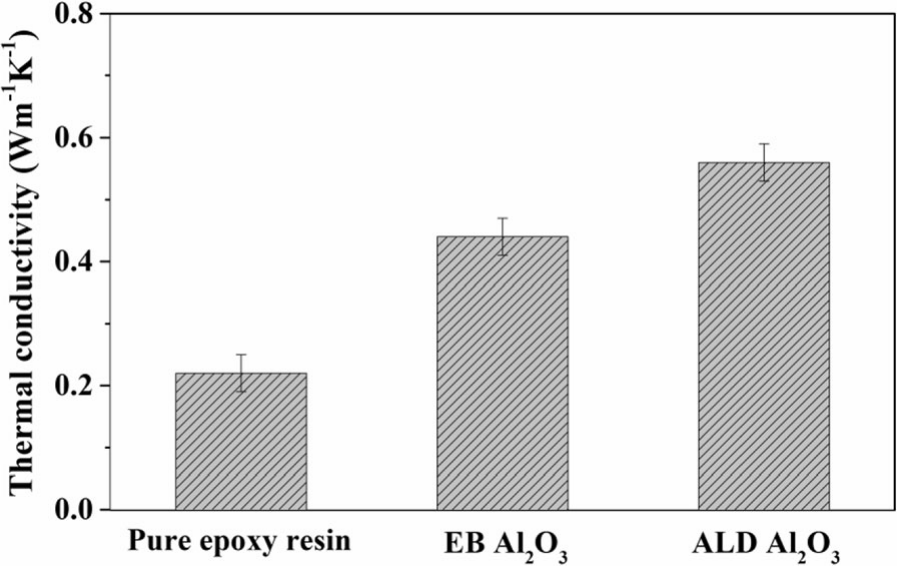
Thermal conductivity analysis of different films: the film with pure epoxy resin and the composite films with VACNTs grown on EB and ALD Al_2_O_3_

Figure 8a, b shows the cross-sectional SEM images of the composite films prepared by filling the matrix in VACNTs. The VACNTs and matrix were fully contacted, and the VACNT-based composite films were successfully synthesized. Their longitudinal thermal conductivities were subsequently analyzed, as shown in Fig. 9. Compared with the pure epoxy resin, VACNTs obviously improved the thermal conductivity of the composite films. In addition, the composite film had higher thermal conductivity with the VACNTs grown on ALD Al_2_O_3_ compared with that on EB Al_2_O_3_. Generally, the thermal conductivity of epoxy resin was much lower than that of multiwall carbon nanotubes, whose experimental thermal conductivity has been reported to be greater than 3000 W m^−1^ K^−1^ at room temperature [37]. Each carbon nanotube was a pathway of thermal dissipation in composite films, and a higher thermal conductivity means more pathways of thermal dissipation. The results indicate that a larger quantity of carbon nanotubes and more dense VACNTs could be achieved on ALD Al_2_O_3_. Commonly, each catalyst nanoparticle could produce at most one carbon nanotube, and the catalyst nanoparticle count might provide an upper limit prediction of the density of VACNTs [35, 38]. However, not all of the catalyst nanoparticles could achieve the formation of a carbon nanotube because the activation energy must be overcome for its nucleation and initial growth. Although the EB Al_2_O_3_ contained a greater number of catalyst nanoparticles than ALD Al_2_O_3_, as mentioned in Fig. 4e, the number of carbon nanotubes on EB Al_2_O_3_ was still less than that on ALD Al_2_O_3_. This result might be explained by a lower activation energy for the nucleation and initial growth of VACNTs on ALD Al_2_O_3_, as shown in Fig. 7. Therefore, in addition to the number of catalyst nanoparticles, the density of VACNTs was still largely dependent on the activation energy for their nucleation and initial growth.

## Conclusions

In this study, we investigated the growth of VACNTs on different oxide buffer layers and their possible growth mechanism. The lifetime of catalyst nanoparticles and the thickness of prepared VACNTs were largely dependent on the diameter and density of the nanoparticles after annealing. The smallest diameter and highest density of nanoparticles were achieved on EB Al_2_O_3_, and the thickest VACNTs were also prepared on this substrate. Conversely, the largest diameter and lowest density of nanoparticles were achieved on sputtered Al_2_O_3_, and almost no VACNTs were prepared on it. These observations might be explained by serious Ostwald ripening of catalyst nanoparticles on sputtered Al_2_O_3_. Compared with EB and ALD Al_2_O_3_, the prepared VACNTs were much thinner on SiO_2_, which might be related to the subsurface diffusion of Fe. In addition, the surface roughness of buffer layers largely influenced the diameter and density of catalyst nanoparticles. Compared with the surface of sputtered Al_2_O_3_, the rough surface of EB Al_2_O_3_ favored a small diameter and high density of catalyst nanoparticles.

Furthermore, the growth of VACNTs was largely dependent on the deposition temperature. At a temperature above 600 °C, the growth rate of VACNTs apparently decreased, which might be caused by serious Ostwald ripening of catalyst nanoparticles, reducing their lifetime. Compared with the activation energy on EB Al_2_O_3_, that on ALD Al_2_O_3_ was much lower, suggesting that the nucleation and initial growth of VACNTs were more easily achieved on it. This lower activation energy might result in more dense VACNTs on ALD Al_2_O_3_, which was confirmed by the higher longitudinal thermal conductivity of the composite film including them. Therefore, in addition to the number of catalyst nanoparticles, the activation energy for the nucleation and initial growth of VACNTs still strongly influenced their density.

## Abbreviations

AFM: Atomic force microscopy
ALD: Atomic layer deposition
CVD: Chemical vapor deposition
DSC: Differential scanning calorimeter
EB: Electron-beam
FESEM: Field-emission scanning electron microscopy
LFA: Laser flash thermal analyzer
RBMs: Radial breathing modes
RMS: Root-mean-square
TEM: Transmission electron microscopy
TMA: Trimethylaluminum
VACNTs: Vertically aligned carbon nanotubes
XPS: X-ray photoelectron spectroscopy
XRR: X-ray reflectivity
